# Minimal change glomerular disease associated with solid neoplasms: a systematic review

**DOI:** 10.1007/s40620-024-02084-6

**Published:** 2024-10-01

**Authors:** Domenico Cozzo, Francesca Orlando, Mariolina Bruno, Adam Ogna, Valentina Forni Ogna

**Affiliations:** 1 Servizio di nefrologia, EOC Ospedale “La Carità”, Locarno, Switzerland; 2 Servizio di medicina interna, EOC Ospedale “La Carità”, Locarno, Switzerland; 3 Faculty of Biomedical Sciences, Università Della Svizzera Italiana, Lugano, Switzerland

**Keywords:** Minimal change disease, Glomerulopathy, Solid neoplasm, Paraneoplastic

## Abstract

**Background:**

Paraneoplastic minimal change disease (MCD) has been associated with hematological malignancies, whereas solid malignancies are commonly associated with membranous glomerulonephritis. In this systematic review of the literature, we describe the clinical features, treatment and outcome of MCD associated with solid neoplasms.

**Methods:**

We performed a systematic review of the MEDLINE, COCHRANE, EMBASE and SCOPUS databases, including case reports of adult patients with biopsy-proven MCD and solid malignancy, without language or time restrictions.

**Results:**

Sixty-seven papers were included, presenting 86 cases with a mean age of 57.8 ± 14.7 years; 41.0% were women. Nephrotic syndrome was the initial presentation in 96.2% of patients; 67.2% had kidney function impairment, and 21.2% required kidney replacement therapy. The most frequent malignancies were malignant thymoma (34.9%), kidney (14.0%), lung (12.8%), and gastrointestinal tumors (12.8%). In 40.7% of cases, the neoplasm diagnosis preceded MCD by 33.8 ± 46.1 months, while in 31.4%, it followed diagnosis of MCD by 12.4 ± 22.6 months. In 27.9%, the neoplasm and kidney disease were diagnosed simultaneously. Immunosuppressive therapy was started in 79.1% of cases and tumor-specific treatment in 83.7%. Remission of MCD was achieved in 80.2% of patients: 38.2% responded to immunosuppressive treatment alone and 29.6% to oncological treatment alone.

**Conclusions:**

The association between MCD and solid neoplasms is well-documented. Immunosuppressive therapy alone induced nephrotic syndrome remission in over one-third of cases; most others responded to tumor-specific treatment. Solid tumor screening should be considered in MCD independently of the steroid response, though more data on solid tumor-associated MCD prevalence are needed for a definitive statement.

**PROSPERO trial registration number:**

CRD42024521854.

**Graphical abstract:**

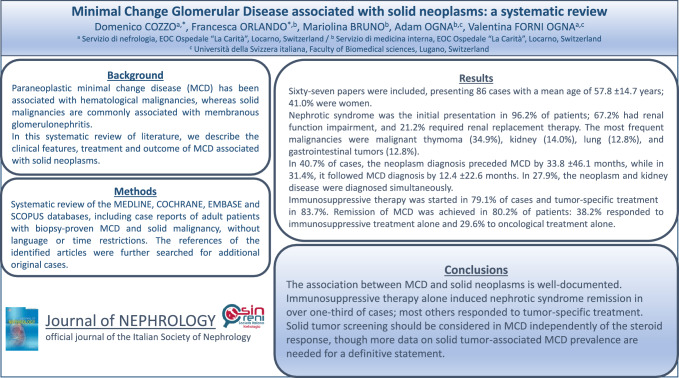

**Supplementary Information:**

The online version contains supplementary material available at 10.1007/s40620-024-02084-6.

## Introduction

Minimal change disease (MCD) is a glomerular disease characterized by minimal histological changes in the kidney, requiring electron microscopy examination of the kidney biopsy to be proven. Its main clinical manifestation is nephrotic syndrome, a clinical condition characterized by severe proteinuria, edema, hypoalbuminemia, and hyperlipidemia [1].

Although MCD is the most common cause of nephrotic syndrome in children, it also occurs in adults and accounts for 6–25% of adult nephrotic syndrome [2–4].

The etiology of MCD is not fully understood, but it is thought to involve both immunological dysregulation and podocyte dysfunction [5]. MCD is most often primary (idiopathic), but can also be secondary to different conditions such as infections, drugs, autoimmune diseases and neoplasms [6, 7]. Among paraneoplastic glomerulopathies, MCD has been reported predominantly in association with hematological malignancies, whereas solid tumors are commonly associated with membranous glomerulonephritis [8, 9]. The observed association between MCD and hematological disorders has been attributed to circulating tumor-secreted cytokines or other leukocyte-derived factors that affect glomerular function. In contrast, the pathophysiological mechanisms underlying glomerulopathies associated with solid tumors remain relatively unclear [8, 9].

Specific recommendations regarding the management of paraneoplastic glomerulopathies are lacking [10]. Unresponsiveness to corticosteroids has been suggested as an indication prompting us to actively search for an underlying malignancy. The rationale behind this recommendation is the assumption that paraneoplastic MCDs may not respond well to immunosuppressive therapy alone due to a possibly different pathophysiology of these conditions [8]. Furthermore, to date, high quality scientific evidence on this specific topic is not available.

In this paper we present a systematic review of the literature focusing on the clinical features, treatment response and outcome of MCD associated with solid malignancies.

## Methods

A systematic review was performed with reference to the Cochrane Handbook for Systematic Reviews of Interventions and reported following the Preferred Reporting Items for Systematic Reviews and Meta-Analyses (PRISMA) guidelines. The protocol was registered In PROSPERO (CRD42024521854).

### Search strategy

A systematic literature search was performed using the MEDLINE, SCOPUS, EMBASE and Cochrane Library databases on December 2023. The search strategy was not limited to any language or year of publication; a specific search string was developed for each database. We used the string “((minimal change disease) OR (glomerulopat*)) AND ((cancer OR tumor OR paraneoplastic) AND (solid))” for **MEDLINE**, the string “('minimal change disease'/exp OR 'minimal change disease') AND ('solid tumor'/exp OR 'solid tumor')” for **EMBASE**, the string “ALL("Minimal change disease" OR "Minimal change glomerulopathy") AND ALL (" tumor" OR "cancer" OR "paraneoplastic") AND ALL ("solid")” for **SCOPUS** and the string “minimal change AND (glomerulopat* OR disease) AND (cancer OR tumor OR paraneoplas*)” for the **Cochrane Library**.

### Study selection

Two investigators (DC and AO) independently screened each citation. Every publication considered potentially relevant by either reviewer was retrieved for a full-text review. Disagreements in study inclusion were resolved by consensus with a third investigator (VFO).

In case of review articles, the original articles describing the reported cases were searched.

The inclusion criteria were: articles reporting original cases or case series, including patients 18 years of age or older, with kidney biopsy-proven MCD and the presence of a solid neoplasm, regardless of the time-correlation to MCD. Exclusion criteria were: full-text article not available, article written in a language not known by the authors and benign tumors.

**Fig. 1:**
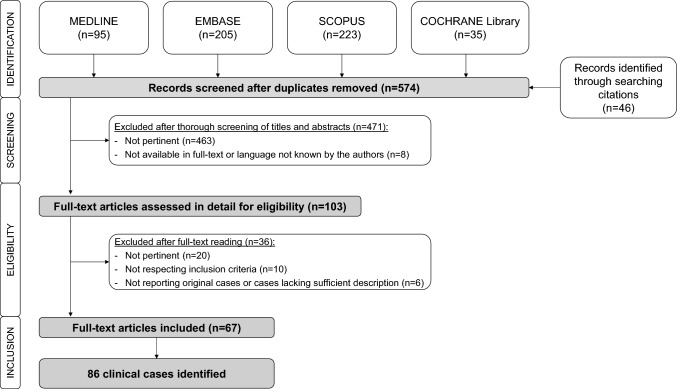
PRISMA flow diagram

We identified a total of 86 original clinical cases from the literature, which were described in a total of 67 papers, published over a time span of 58 years [12–78]. The PRISMA flow diagram is presented in Fig. 1.

### Data extraction

Extraction of data from eligible articles was performed using a standardized data collection form. The following data were extracted: age, gender, baseline creatinine value, plasma albumin and urine-24 h protein peak. Concerning kidney disease, we identified the following clinical presentations: nephrotic syndrome (defined as proteinuria > 3.5 g/24 h, hypoalbuminemia < 30 mg/l and/or anasarca) and functional kidney impairment (defined as baseline creatinine value > 120 µmol/l or described acute kidney failure and/or need for kidney replacement therapy). Concerning MCD disease, we collected: potential confounding factors (i.e. other causes potentially triggering MCD), the time interval between tumor and MCD diagnosis, administration of MCD-related therapy (including glucocorticosteroids and/or non-steroidal immunomodulating therapy), and lastly, kidney disease outcome and timing (after immunosuppressive therapy and/or after oncological treatment).

Positive kidney outcome (“response to treatment”) was considered every report of “partial response” or “complete response” in the original articles, since most of the articles described neither the exact definition of these terms nor the values of proteinuria in the follow-up.

We collected tumor type, staging, tumor treatment modalities (chemotherapy and/or radiotherapy and/or surgery) and tumor outcome.

### Quality of the included studies

The quality of the included articles was independently assessed by two investigators (DC and FO) using the pertinent items of the Joanna Briggs Institute (JBI) Critical Appraisal Tools for Case reports/Case series [11], resulting in a score between 0 and 5 points. Scores of 4 and 5 were interpreted as ‘high quality’, 2–3 as ‘intermediate quality’ and 0–1 as ‘low quality’. Most of the included articles were judged as having high (*N* = 19, 28.4%) or intermediate quality (*N* = 42, 62.7%), while only six case reports showed low quality of data presentation. The quality assessment of the included papers is reported in Supplementary Table S1.

### Statistical analysis

Statistical analysis was conducted using R for Windows, version 4.3.1 (R Foundation for Statistical Computing, Vienna, Austria). We calculated descriptive statistics for the population. Data are presented as number of available results, mean value ± standard deviation for continuous variables, and absolute numbers and percentages of available observations for categorical variables. We compared the characteristics of two groups—“malignant thymoma” and “other solid tumors”—using Student’s -t test and Fisher’s exact test, as appropriate.

## Results

### Clinical findings

We identified a total of 86 original clinical cases from the literature, which were described in 67 papers, published over a time span of 58 years [12–76]. Among the 86 original cases identified, 30 were categorized as belonging to the “malignant thymoma group” and 56 cases into the “other solid tumor group”.

The mean age at presentation was 57.8 ± 14.7 years, with patients with malignant thymoma tumors being significantly younger (52.8 ± 14.0 vs. 60.5 ± 14.5, p = 0.016). Overall, 41% were women, without differences between groups.

The initial presentation was nephrotic syndrome in all patients of the other solid tumors group and in 88.6% of patients with malignant thymoma. Among them, 67.2% had kidney function impairment and 21% required kidney replacement therapy, without significant differences between groups.

A potential confounding iatrogenic factor was identified in only four cases: two patients had been exposed to nonsteroidal anti-inflammatory drugs, one to interferon and one to sorafenib.

The characteristics of the study populations are described in Table 1.

**Table 1:** Study population

	Available data (*N*)	All (*N* = 86)	Malignant thymoma (*N* = 30)	Other solid tumors (*N* = 56)	*p*
Age	85	57.8 ± 14.7	52.8 ± 14.0	60.5 ± 14.5	**0.016**
Female gender	85	41 (48.2)	17/30 (56.7)	24/55 (43.6)	0.267
*Initial nephrologic presentation*					
Functional kidney impairment	61	41 (67.2)	15/19 (78.9)	26/42 (61.9)	0.246
Nephrotic syndrome	79	76 (96.2)	23/26 (88.4)	53/53 (100)	**0.033**
Need for renal replacement therapy	66	14 (21.2)	5/15 (33.3)	9/51 (17.6)	0.279
Creatinine (µmol/l)	62	187.9 ± 169.7	205.1 ± 216.7	174.7 ± 124.0	0.520
Plasma albumin (g/l)	71	19.7 ± 7.3	20.0 ± 8.4	19.5 ± 6.6	0.779
24-h proteinuria (g/24 h)	78	12.02 ± 8.87	15.0 ± 11.5	10.3 ± 6.5	0.054
*Tumor-related characteristics*					
Staging	59				0.387
Localized		18 (30.5)	8/21 (38.1)	10/38 (26.3)	
Metastatic		31 (52.5)	13/21 (61.9)	28/38 (73.7)	
*Timing of tumor diagnosis with respect to kidney disease*	86				**0.002**
Before		35 (40.7)	20/30 (66.7)	15/56 (26.7)	
Timing (months)		33.8 ± 46.1	49.8 ± 54.2	11.1 ± 12.0	**0.005**
Simultaneous		24 (27.9)	4/30 (13.3)	20/56 (35.7)	
After		27 (31.4)	6/30 (20)	21/56 (37.5)	
Timing (months)		12.4 ± 22.6	37.0 ± 38.2	5.4 ± 7.9	0.099

Data presented as mean ± SD or *N* (%), *p* < 0.05 is highlighted with bold font

**Fig. 2:**
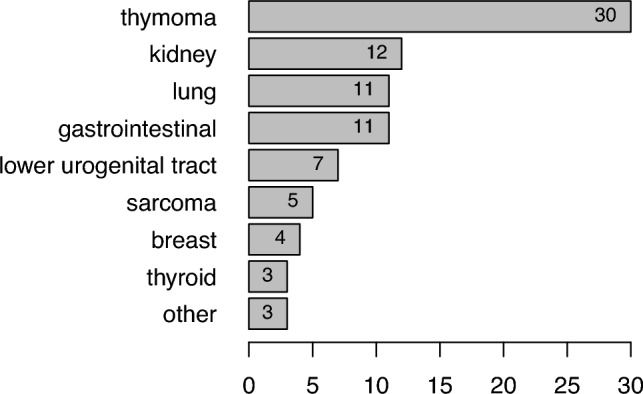
Relative frequency of reported tumors

Thymic tumors were the most frequent malignancy (34.9%), followed by kidney (14.0%), lung (12.8%) and gastrointestinal tumors (12.8%). At diagnosis, 52.5% of tumors were in the metastatic stage. The characteristics of the reported neoplasms are described in Table 1 and in Fig. 2.

In 40.7% of cases, the neoplasm diagnosis preceded MCD diagnosis by 33.8 ± 46.1 months, while in 31.4% of cases, tumor diagnosis followed MCD diagnosis by 12.4 ± 22.6 months; in the remaining cases (27.9%), the tumor was diagnosed simultaneously with the onset of kidney disease. In the malignant thymoma group, the tumor diagnosis mostly preceded the glomerulopathy (in 60.7% of cases vs. 26.7% of cases in the non-malignant thymoma group, *p* = 0.002) by a larger time frame (49.8 ± 54.2 vs. 11.1 ± 12.0 months, *p* = 0.005).

### Treatments and outcomes

Two main treatment modalities were administered to the reported patients: (1) kidney-related therapy, including steroids and non-steroidal immunomodulating drugs; (2) tumor-related therapy, including surgery, chemotherapy, and/or radiotherapy.

Concerning kidney-related therapy, immunosuppressive therapy was started in 79.1% of cases (in 76.7% of cases including a steroid). The other agents used were cyclophosphamide (*N* = 12), calcineurin inhibitors (*N* = 7), monoclonal anti-CD20 antibody (*N* = 1) and azathioprine (*N* = 2).

Malignant thymoma patients were more frequently treated with a combination of steroids and a second immunosuppressive agent—mostly cyclophosphamide—compared to patients affected by other tumors (46.7% vs. 7.1%, p < 0.001).

In the majority of cases (83.7%), a tumor-specific treatment was started. Combined surgery plus chemo-radiotherapy was more frequently used in the group of thymoma patients (60.0% vs. 19.6%, *p* < 0.001) than in the other group.

A favorable outcome of the glomerulopathy was achieved in 80.2% of patients (67.9% of patients with malignant thymoma and 86.8% patients with other neoplasms): 38.2% of patients responded to immunosuppressive treatment alone, 29.6% after oncological treatment alone and 11.1% after the combination of immunosuppressive and tumor-related therapy, whilst 1.2% showed spontaneous remission. Patients with malignancies other than thymoma more often showed kidney disease response after tumor-targeted therapy alone (39.6% vs. 10.7%, p = 0.010).

Concerning tumor outcome, half of patients completely or partially responded to tumor-specific therapy, without significant differences between groups.

Data concerning therapies and outcomes are summarized in Table 2.

**Table 2:** Treatment and outcomes

	All (*N* = 86)	Malignant thymoma (*N* = 30)	Other solid tumors (*N* = 56)	*p*
*Kidney-related therapy*				
Available data (*N*)	86	30	56	
Only steroids	48 (55.8)	13 (43.3)	35 (62.5)	0.112
Only non-steroidal immunomodulating therapy	2 (2.3)	0	2 (3.6)	0.540
steroids + non-steroidal immunomodulating therapy	18 (20.9)	14 (46.7)	4 (7.1)	**< 0.001**
No treatment	18 (20.9)	3 (10.0)	15 (26.8)	0.096
*Tumor-related therapy*				
Available data (N)	86	30	56	
Surgery alone	27 (31.4)	5 (16.7)	22 (39.3)	**0.050**
Chemo-radiotherapy alone	16 (18.6)	5 (16.7)	11 (19.6)	1.00
Surgery + chemo-radiotherapy	29 (33.7)	18 (60.0)	11 (19.6)	** < 0.001**
No treatment	14 (16.3)	2 (6.7)	12 (21.4)	0.124
*Kidney outcome*				
Available data (*N*)	81	28	53	
Response after steroids alone	27 (33.3)	10 (35.7)	17 (32.1)	0.806
Response after steroids + non-steroidal immunomodulating therapy	4 (4.9)	3 (10.7)	1 (1.9)	0.117
Response after tumor-related therapy alone	24 (29.6)	3 (10.7)	21 (39.6)	**0.010**
Response after both immunosuppression and tumor-related therapy	9 (11.1)	3 (10.7)	6 (11.3)	1.00
Spontaneous response	1 (1.2)	0	1 (1.9)	1.00
Non responders	16 (19.8)	9 (32.1)	7 (13.2)	0.075
*Tumor outcome*				
Available data (*N*)	54	14	40	
Complete remission	21 (38.9)	4 (28.6)	17 (42.5)	0.526
Partial remission	6 (11.1)	4 (28.6)	2 (5.0)	**0.033**
Progression/relapse	27 (50.0)	6 (42.9)	21 (52.5)	0.757

Data presented as *N* (%), *p* < 0.05 is highlighted with bold font

## Discussion

Our systematic review confirms an association between MCD and solid neoplasms, that is consistently described in the literature, challenging the preferential associations between MCD and hematological malignancies only, and between solid tumors and membranous glomerulonephritis only [53].

By collecting a population of 86 patients—the largest described to date—we were able to highlight some important aspects of MCD associated with solid neoplasms. Firstly, malignant thymoma is the most common neoplasm described in association with MCD (39.4%), however, a variety of solid neoplasms have been reported, particularly kidney, lung and gastrointestinal tumors. In addition, the present results show that immunosuppressive treatment alone can positively influence renal outcome in 38.2% of cases. It is important to note that response to steroid treatment is not a way of distinguishing between idiopathic and paraneoplastic forms of MCD. The response rate to immunosuppressive drugs suggests a key role for the immune system in the pathogenesis of paraneoplastic MCD.

The association between glomerular disease and thymic tumors was first documented in 1980 [79]. However, the incidence of this association may be quite low, as the occurrence of nephropathy is rarely reported in retrospective surgical series.

In 2005, Karras et al. published the largest retrospective case series of glomerulopathies associated with thymic disease, collected in a multicenter study over a 22 year period [50]. Of the 21 patients identified, 19 had high-grade malignant thymoma and two had non-malignant thymic hyperplasia. The most common glomerular disease identified by kidney biopsy was MCD (14 out of 21 cases). According to the authors, the predominance of one type of glomerular disease suggests that MCD is not a coincidental event in patients with thymic disease.

Four criteria have been proposed by Ronco et al. to support the diagnosis of paraneoplastic glomerulopathy [80]: (1) the absence of obvious alternative etiologies for the glomerulopathy; (2) the existence of a temporal relationship between the diagnosis of the glomerulopathy and the cancer; (3) clinical (and histological) remission after complete surgical removal of the tumor or complete remission achieved by chemotherapy; (4) recurrence of the tumor associated with an increase in associated symptoms.

When analyzing the cases in the present review, we found that: (1) only four patients were exposed to drugs potentially triggering MCD before the onset of the nephrotic syndrome; (2) the time interval between tumor diagnosis and diagnosis of MCD was relatively short in the non-thymoma group, whereas the time span was longer among the patients in the malignant thymoma group. This discrepancy could be attributed to the study design of the largest available series, which featured a retrospective search for MCD after a long follow-up of thymoma patients, thus allowing for the detection of cases with a distant temporal association. In nearly half (14 out of 30) of the thymoma patients, the tumor diagnosis preceded MCD by more than one year; this did not seem to be associated with thymoma relapse, that was reported in only three cases. The authors propose that dysregulation of the immune system following thymoma and/or thymectomy may be the possible underlying mechanism explaining the causal link despite the extended time span, similar to other parathymic syndromes such as pure red cell aplasia, myasthenia gravis or pemphigus [50]; (3) the tumor-related treatment alone allowed for partial or complete remission of the glomerular disease in 29.6% of patients (39.6% in the non-thymoma group), while the combination of kidney- and tumor-treatment led to remission in 79.0% of patients; (4) recurrence of nephrotic syndrome after tumor relapse has only been described in one case of lung tumor-associated MCD [22]. However, it is important to note that in some cases the two conditions may evolve independently [53].

The observed response to immunosuppressive drugs suggests a key role for the immune system in the pathogenesis of paraneoplastic MCD. The precise pathophysiological mechanism underlying the association between solid tumors and MCD remains to be elucidated, although several studies have investigated potential mechanisms, particularly in thymic tumors, where an immune dysregulation appears to be central.

As the primary site for T lymphocyte maturation via positive and negative selection, the thymus plays a critical role in the development of autoimmune disease, with an imbalance between autoreactive lymphocytes and regulatory mechanisms. Disturbances in the proportion of circulating lymphocytes are present in individuals with thymoma [81]. It has been suggested that thymoma-associated T-cell lymphoproliferation may induce aberrant immune responses, possibly involving the production of cytokines or growth factors, which, in turn, affect the glomerular barrier.

The well-established association between MCD and malignant lymphoma further supports the notion that T cell-mediated immunity is involved in MCD [8].

In addition, an experimental animal model, the Buffalo/Mna rat thymoma model, which spontaneously develops MCD/Focal Segmental GlomeruloSclerosis (FSGS) and myasthenia gravis-like disease in association with the thymic disease, strengthens the pathophysiological link [82].

Interestingly, the present results show that immunosuppressive treatment alone positively influenced the kidney outcome in 38.2% of cases, with no difference between patients with thymic neoplasm and other tumors. This suggests a widespread role of the immune system in the mechanisms of selective proteinuria associated with this disease. The pathophysiology underlying paraneoplastic glomerulopathies is thought to involve a variety of immunological mechanisms, such as cytokines, growth factors or tumor antigens, all of which can be effectively attenuated by immunosuppressive interventions [53].

A wide range of immunological abnormalities have been documented in cases of paraneoplastic MCD, affecting both humoral and cell-mediated immune responses. MCD may be triggered by cytokines released by tumor-infiltrating lymphocytes and macrophages, resulting in increased glomerular permeability [53]. Disorders in the balance between Th1 and Th2 subsets within circulating lymphocyte populations, together with abnormal T-cell responses [83], have been observed in patients with idiopathic nephrotic syndrome. It is therefore conceivable that lymphoid tissues may be involved in both systemic and glomerular responses induced by solid tumors. The initial tumor-induced inflammation may progress to granulomas, accompanied by cytokine secretion, which in turn leads to increased permeability of the glomerular basement membrane.

Some circulating factors, i.e. vascular endothelial growth factor (VEGF), have also been linked to cancer-associated MCD: in a case report of MCD associated with colon cancer [48], the development of glomerular disease was linked to VEGF levels, and tumor excision provoked a decrease in VEGF levels and prompt remission of the nephrotic syndrome. Involvement of VEGF in the pathogenesis of MCD has also been hypothesized in a few cases of malignant mesothelioma [63]. In the kidney, VEGF-A is expressed by both podocytes and renal tubular epithelial cells and VEGF signaling seems to be critical for maintaining glomerular membrane permeability [84].

The pro-angiogenic effect of VEGF is primarily mediated by the endothelial VEGF receptor 2 (VEGFR2), a tyrosine kinase receptor [85]. Tyrosine kinase inhibitors interfere with the activity of one or more families of receptor tyrosine kinases (including VEGFR2) and are increasingly used as anti-cancer therapy, for example in advanced renal cell carcinoma. Nephrotoxic complications associated with tyrosine kinase inhibitors, particularly hypertension and proteinuria, have become a major concern, with biopsy specimens from patients with nephrotic syndrome on tyrosine kinase inhibitors showing podocytopathies, MCD and collapsing FSGS [86].

In our systematic review, we found only one patient with renal cell carcinoma treated with the tyrosine kinase inhibitor sorafenib who was diagnosed with MCD.

During the last KDIGO Controversies Conference on onco-nephrology, the expert panel suggested screening for neoplasms in patients with MCD and unexplained anemia, abnormal serum protein electrophoresis, hepatosplenomegaly, or lymphadenopathy, thus focusing on the search for an associated hematologic neoplasm [87]. Based on the findings of our study, screening of MCD patients should also include solid tumors, though a conclusive recommendation necessitates further data on the prevalence of solid tumor-associated MCD. Screening should be conducted even in MCD patients who respond well to steroids alone.

In the absence of internationally approved guidelines for MCD, a tumor screening protocol similar to the one recently proposed by an expert panel for patients with nephrotic syndrome could be applied, including comprehensive clinical history and physical examination, blood tests with complete blood count, kidney and liver function tests, a chest CT scan and a complete abdominal ultrasound or abdominal CT scan. This screening regimen would identify most tumors reported in our case review. Age- and country-specific tumor screening examinations, such as mammography, prostate cancer screening, fecal occult blood testing or colonoscopy, should also be performed [9]. In addition, a high degree of clinical suspicion for underlying malignancies should be maintained during the follow-up of MCD patients.

Once cancer is diagnosed, prioritizing cancer treatment as the primary intervention before starting steroid therapy could be an effective strategy, as MCD may improve following treatment of the underlying malignancy. Such a strategy would reduce the need for administering high-dose glucocorticoids and spare patients the occurrence of steroid-associated adverse effects. On the other hand, corticosteroids may be a treatment option with a high likelihood of efficacy on renal outcomes in cases of severe kidney disease or when rapid access to oncological treatment is not possible.

Although this is the largest systematic study describing the association between solid tumors and MCD, it has several limitations: the study design does not allow us to assess the prevalence of paraneoplastic MCD, and the small number of published cases over 58 years suggests a possible relevant rate of under-reporting. Another limitation is the presence of publication bias, as pre-existing case series focusing on thymic tumors may have influenced the prominence of thymic tumors as the most frequently associated tumor with paraneoplastic MCD in our study.

In conclusion, a solid malignancy may have an unusual onset manifestation such as MCD nephrotic syndrome. MCD may be one of the consequences of the immune dysregulation associated with solid tumors, and the response to steroids consistently observed in the literature supports the hypothesis of underlying immunological mechanisms that need to be elucidated in the future.

The management of paraneoplastic MCD is complex and requires an individualized multidisciplinary approach, entailing treatment of the underlying malignancy, but also considering a kidney-targeted therapy.

## Supplementary Information

Below is the link to the electronic supplementary material.

Supplementary file1 (DOCX 20 KB)

## Data Availability

All the study data were collected from the publicly available cited original works, available on the MEDLINE, COCHRANE, EMBASE and SCOPUS repositories.
