# Supplementary material for: Minimal change glomerular disease associated with solid neoplasms: a systematic review
Source: J Nephrol. 2024 Oct 1;38(2):343–52. doi: 10.1007/s40620-024-02084-6 (PMC11961479; doi:10.1007/s40620-024-02084-6)
Supplement: Supplementary file 1 — Supplementary file1 (DOCX 20 KB) [file 40620_2024_2084_MOESM1_ESM.docx]

**Supplementary Table S1: quality assessment of the included articles**

| **Author** | **Year** | **Ref.** | **Original cases (N)** | **Article quality** |
| --- | --- | --- | --- | --- |
| Lee JC et al. | 1966 | 11 | 1 | Low |
| Banerji LG et al. | 1970 | 12 | 1 | High |
| Caruana RJ et al. | 1980 | 13 | 1 | High |
| Hulter HN et al. | 1980 | 14 | 1 | Intermediate |
| Varsano S et al. | 1980 | 15 | 1 | Intermediate |
| Tan YO et al. | 1982 | 16 | 1 | High |
| Moorthy AV et al. | 1983 | 17 | 2 | Intermediate |
| Hillion D et al. | 1983 | 18 | 1 | Intermediate |
| Scadding GK et al. | 1983 | 19 | 1 | Intermediate |
| Schroeter NJ et al. | 1986 | 20 | 1 | Intermediate |
| Singer C et al. | 1986 | 21 | 1 | Intermediate |
| Hirokawa M et al. | 1986 | 22 | 1 | Low |
| Whelan T et al. | 1988 | 23 | 1 | Intermediate |
| Rapoport J et al. | 1989 | 24 | 1 | High |
| Chan PC et al. | 1990 | 25 | 2 | Intermediate |
| Meyrier A et al. | 1992 | 26 | 2 | Intermediate |
| Lee SJ et al. | 1992 | 27 | 1 | Intermediate |
| McDonald P et al. | 1992 | 28 | 1 | Intermediate |
| Ogawa M et al. | 1992 | 29 | 1 | Intermediate |
| Martinez-Vea A et al. | 1993 | 30 | 4 | Intermediate |
| Abouchacra S et al. | 1993 | 31 | 1 | Intermediate |
| Gallego E et al. | 1994 | 32 | 1 | Low |
| Woodrow G et al. | 1995 | 33 | 1 | Intermediate |
| Gandini E et al. | 1996 | 34 | 1 | Intermediate |
| Tashiro M et al. | 1996 | 35 | 1 | Low |
| Ishida I et al. | 1996 | 36 | 2 | Intermediate |
| Cabezuelo JB et al. | 1996 | 37 | 1 | High |
| Sood AK et al. | 1997 | 38 | 1 | Intermediate |
| Schillinger F et al. | 1997 | 39 | 1 | Intermediate |
| Auguet T et al. | 1998 | 40 | 1 | Intermediate |
| Zinger C et al. | 1998 | 41 | 1 | Intermediate |
| Lasseur C et al. | 1999 | 42 | 1 | Intermediate |
| Macanovic M et al. | 2000 | 43 | 1 | Intermediate |
| Lee HC et al. | 2001 | 44 | 1 | Intermediate |
| Stuart S et al. | 2001 | 45 | 1 | High |
| Farmer CKT et al. | 2001 | 46 | 1 | Intermediate |
| Taniguchi K et al. | 2004 | 47 | 1 | Intermediate |
| Ryu DR et al. | 2004 | 48 | 1 | High |
| Karras A et al. | 2005 | 49 | 12 | High |
| Han SS et al. | 2008 | 50 | 1 | Intermediate |
| Teoh DC et al. | 2008 | 51 | 1 | High |
| Bacchetta J et al. | 2009 | 52 | 1 | High |
| Bonkain F et al. | 2010 | 53 | 1 | Intermediate |
| Sherman JL et al. | 2010 | 54 | 1 | High |
| Li JYZ et al. | 2010 | 55 | 1 | Low |
| Naritakaya Y et al. | 2010 | 56 | 1 | Intermediate |
| Gonzàlez-Fontal GR et al. | 2011 | 57 | 1 | Intermediate |
| Tsukamoto Y et al. | 2015 | 58 | 1 | High |
| Gharwan H et al. | 2015 | 59 | 1 | High |
| Lotlikar R. et al. | 2015 | 60 | 1 | Intermediate |
| San A et al. | 2015 | 61 | 1 | Intermediate |
| Yildiz H et al. | 2016 | 62 | 1 | Intermediate |
| Gurram S at al. | 2017 | 63 | 1 | Intermediate |
| Soliman K et al. | 2018 | 64 | 1 | Intermediate |
| Montague E et al. | 2019 | 65 | 1 | Intermediate |
| Khurana A et al. | 2019 | 66 | 1 | High |
| Ngoh CLY et al. | 2019 | 67 | 1 | High |
| Pappy L et al. | 2019 | 68 | 1 | Intermediate |
| Yasui Y et al. | 2020 | 69 | 1 | High |
| Dang S et al. | 2020 | 70 | 1 | Low |
| Masuda S et al. | 2021 | 71 | 1 | Intermediate |
| Ihara K et al. | 2021 | 72 | 1 | High |
| Romero-Castellanos WO. et al. | 2021 | 73 | 1 | Intermediate |
| Nakano Y et al. | 2022 | 74 | 1 | Intermediate |
| Yin J et al. | 2023 | 75 | 2 | High |
| Cai X et al. | 2023 | 76 | 1 | Intermediate |
| Cozzo D et al. | 2024 | 77 | 1 | High |
